# Retraction Note: Brace classification study group (BCSG): part one – definitions and atlas

**DOI:** 10.1186/s13013-018-0171-1

**Published:** 2018-11-07

**Authors:** Theodoros B. Grivas, Jean Claude de Mauroy, Grant Wood, Manuel Rigo, Michael Timothy Hresko, Tomasz Kotwicki, Stefano Negrini

**Affiliations:** 1 Department of Orthopedics and Traumatology, “Tzaneio” General Hospital, Piraeus, Greece; 2 Department of Orthopaedic Medicine, Clinique du Parc, 155, Bd Stalingrad, 69006 Lyon, France; 3 Align Clinic, San Mateo, CA USA; 4 Institute Elena Salvá, Barcelona, Spain; 5 Harvard University, Boston Children’s Hospital, Boston, MA USA; 6 University of Medical Sciences, Poznan, Poland; 7 Department of Clinical and Experimental Sciences, University of Brescia, Brescia, Italy; 8 Don Gnocchi Foundation, Milan, Italy

## Retraction Note

The Publisher has retracted this article [1], as it is unclear whether written informed consent was obtained from some patients to publish their images in an Open Access journal. The article is no longer available online in order to protect the privacy of the individuals. GW, SN, JCDM and TG disagree with this retraction. MR, MTH and TK did not state whether they agreed or disagreed with the retraction.
